# Survival nomogram for melanoma with bone metastasis based on the SEER database and an external validation cohort

**DOI:** 10.3389/fonc.2025.1680191

**Published:** 2025-11-06

**Authors:** Yuping Lu, Lizhu Chen, Shicheng Zheng, Li Zhu, Zhida Wu, Yu Chen, Jing Lin

**Affiliations:** 1 Department of Medical Oncology, Clinical Oncology School of Fujian Medical University, Fujian Cancer Hospital, Fuzhou, Fujian, China; 2 Cancer Bioimmunotherapy Center, Clinical Oncology School of Fujian Medical University, Fujian Cancer Hospital, Fuzhou, Fujian, China; 3 School of Basic Medical Sciences, Fujian Medical University, Fuzhou, Fujian, China; 4 Department of Pathology, Clinical Oncology School of Fujian Medical University, Fujian Cancer Hospital, Fuzhou, Fujian, China

**Keywords:** melanoma, bone metastasis, SEER, prognosis, nomogram

## Abstract

**Background:**

We aimed to develop and validate a comprehensive prognostic model for melanoma bone metastasis.

**Methods:**

Data on melanoma bone metastasis patients were obtained from the SEER database and Fujian Cancer Hospital. Cox regression analysis was conducted to identify independent prognostic factors and to establish a Nomogram to predict the overall survival rate of patients.

**Results:**

We generated a Nomogram chart incorporating factors such as Age, Site, AJCC T stage, AJCC N stage, Surg Prim, Systemic and Sur Seq, Surg or Rad Seq, DX Brain, DX Liver, DX Distant LN, Tumor number, First malignant primary, Marital status, and Urban. The 1-year, 3-year, and 5-year OS rate AUCs for the training cohort were 0.715, 0.711, and 0.714, respectively, with a c-index of 0.656; the 1-year, 3-year, and 5-year OS rate AUCs for the internal validation cohort were 0.695, 0.725, and 0.719, respectively, with a c-index of 0.650; the 1-year, 3-year, and 5-year OS rate AUCs for the external validation cohort were 0.714, 0.791, and 0.842, respectively, with a c-index of 0.710. Calibration curves showed the consistency between the Nomogram’s observed and predicted prognostic outcomes.

**Conclusion:**

Our model can be used to predict the prognosis of melanoma bone metastasis.

## 1 Introduction

Bone metastasis is one of the common complications of various malignant tumors, especially common in breast cancer, prostate cancer, lung cancer, thyroid cancer, and renal cancer (1). These tumor cells can reach the bones through the bloodstream or lymphatic system and form new tumor foci within the bones. The prognosis of bone metastasis is influenced by various factors, and generally, the higher the degree of malignancy of the primary tumor, the worse the prognosis of bone metastasis is usually.

Melanoma is a malignant tumor that originates from melanocytes. According to the global cancer data from the WHO in 2022, its incidence rate is 1.7% (2). Although treatment methods have been continuously advancing, the median overall survival (OS) is still less than 1.5 years (3). Advanced melanoma is prone to bone metastasis, which destroys bone tissue. Statistics show that 5% to 20% of melanoma patients will encounter metastatic bone disease during the course of their illness (4–6) Compared to those patients whose primary metastatic sites are the skin, distant lymph nodes, or lungs, patients with liver, central nervous system, bone, and multiple distant metastatic sites have significantly shorter survival times (7). Patients with bone metastasis often face severe complications such as hypercalcemia, fractures, and spinal cord compression, and some patients need to rely on local radiotherapy and orthopedic surgery to alleviate pain (6). Several studies have proven that bone metastasis shortens the expected survival of melanoma patients (7, 8).

To effectively address the challenges faced by melanoma patients with bone metastasis, accurate prediction of their prognosis has become key to improving treatment outcomes and extending patients’ survival. However, there is currently a lack of systematic research reports specifically focused on the prognosis of melanoma bone metastasis. Given the precision and intuitive advantages of Nomogram charts in predicting the survival rates of cancer patients, this study aims to identify the key factors affecting the prognosis of melanoma patients with bone metastasis and to construct a scientific and practical Nomogram prognostic model. This model will visually display the relationship between various prognostic factors and OS, providing a powerful tool for clinical practice and personalized treatment.

## 2 Methodology

### 2.1 Data sources and patient selection

The SEER database (http://www.seer.cancer.gov), which stands for the Surveillance, Epidemiology, and End Results Program of the National Cancer Institute in the United States, has been meticulously documenting patient information of various types of cancer from different states and counties in the United States. This includes details such as age, gender, race, year of diagnosis, marital status, number of tumors, distant metastasis, and treatment information. For this study, the SEER*Stat 8.4.3 software was utilized to extract data on patients pathologically diagnosed with melanoma between the years 2000 and 2021. External Validation Cohort: A total of 152 patients diagnosed with melanoma bone metastasis from Fujian Cancer Hospital between 2007 and 2024 were collected as an external validation cohort.

Inclusion criteria: Pathological diagnosis of melanoma. Exclusion criteria: 1. Unknown survival status; 2. Unknown survival time; 3. Patients without bone metastasis; 4. Significant data missing.

### 2.2 Data collection

We collected the following variables from the selected cohorts: Surg Prim (primary site surgery), Scope Reg LN Sur (regional lymph node dissection), Surg or Rad Seq (neoadjuvant or adjuvant radiotherapy), Radiation, Systemic and Sur Seq (neoadjuvant or adjuvant therapy), Ulceration, DX Brain(brain metastasis), DX Liver(liver metastasis), DX Lung(lung metastasis), DX Distant LN(distant lymph node metastasis), DX Other(other metastasis), Age, Sex, Race, Site, American Joint Committee on Cancer Staging System (AJCC) T stage, AJCC N stage, Surg Oth Reg or Dis(other regional or distant metastatic surgery), Chemotherapy, Tumor number, First malignant primary, Marital status, and Urban information. Subsequently, we collected data on months of survival and survival status as the outcome variables. The primary endpoint was OS, which is defined as the time from diagnosis to death due to any cause.

### 2.3 Statistical analysis

Patients included from the SEER database were matched 1:1 based on the presence or absence of bone metastasis using propensity score matching. We selected 23 baseline covariates on the basis of prior literature and clinical relevance: Surg Prim, Scope Reg LN Sur, Surg or Rad Seq, Radiation, Systemic and Sur Seq, Ulceration, DX Brain, DX Liver, DX Distant LN, DX Other, age, sex, race, primary site, AJCC T stage, AJCC N stage, Surg Oth Reg or Dis, chemotherapy, DX Lung, multi-primary status, first malignant primary, marital status and urban residence. A multivariable logistic regression model with bone metastasis (yes/no) as the outcome estimated each patient’s propensity score. Matching was performed 1:1 without replacement using nearest-neighbor matching, with a caliper width of 0.1 × SD of the logit(propensity score). Only patients within the common-support region were retained. Balance was assessed by the standardized mean difference (SMD); all 23 covariates achieved SMD < 0.08 after matching, indicating adequate balance. Descriptive statistics were used for demographic information. Kaplan-Meier analysis was used for survival estimation and comparison of different variables, including median survival time and 95% confidence intervals (95%CI). The log-rank test was used to compare the significance of survival curves. Patients with bone metastasis from melanoma in the SEER data were randomly (7:3) divided into a training group and an internal validation group. Univariate and multivariate Cox regression analyses were performed on the training group, and variables identified as significant in both univariate and multivariate Cox regression analyses were used to generate the Nomogram chart. Hazard ratios (HR) and corresponding 95% CI were calculated for each variable at all levels. The predictive performance of the line chart was estimated using the c-index, receiver operating characteristic(ROC) curve, area under the curve (AUC) and calibration curve for the training group, internal validation group, and external validation cohort. All statistical analyses and chart formations were conducted using SPSS 26.0 and Rstudio software. Statistical significance was set at P < 0.05.

## 3 Results

### 3.1 Basic characteristics of enrolled patients

Based on the inclusion and exclusion criteria, a total of 184,129 patients registered in the SEER database were included, of which 2,358 were patients with bone metastasis of melanoma. A total of 2,358 melanoma patients with bone metastases were ultimately included; concurrently, an additional 2,358 melanoma patients without bone metastases were selected from the SEER database as controls for 1:1 propensity score matching. The basic characteristics of the included patients are shown in **Supplementary Table 1**. There was a statistically significant difference in Survival Status, Surg Prim, Scope Reg LN Sur, Surg or Rad Seq, Radiation, Systemic and Sur Seq, Ulceration, DX Brain, DX Liver, DX Distant LN, DX Other between patients with bone metastasis and those without bone metastasis of melanoma (P<0.05). However, there was no statistically significant difference in Age, Sex, Race, Site, AJCC T stage, AJCC N stage, Surg Oth Reg or Dis, Chemotherapy, DX lung, Tumor number, First malignant primary, Marital status, and Urban (P>0.05). In addition, 126 patients with melanoma combined with bone metastasis from Fujian Cancer Hospital were included as the external validation cohort. The baseline situation of the external validation cohort is shown in **Supplementary Table 2**.

### 3.2 Analysis of survival situation in melanoma with or without bone metastasis

**Figure 1:**
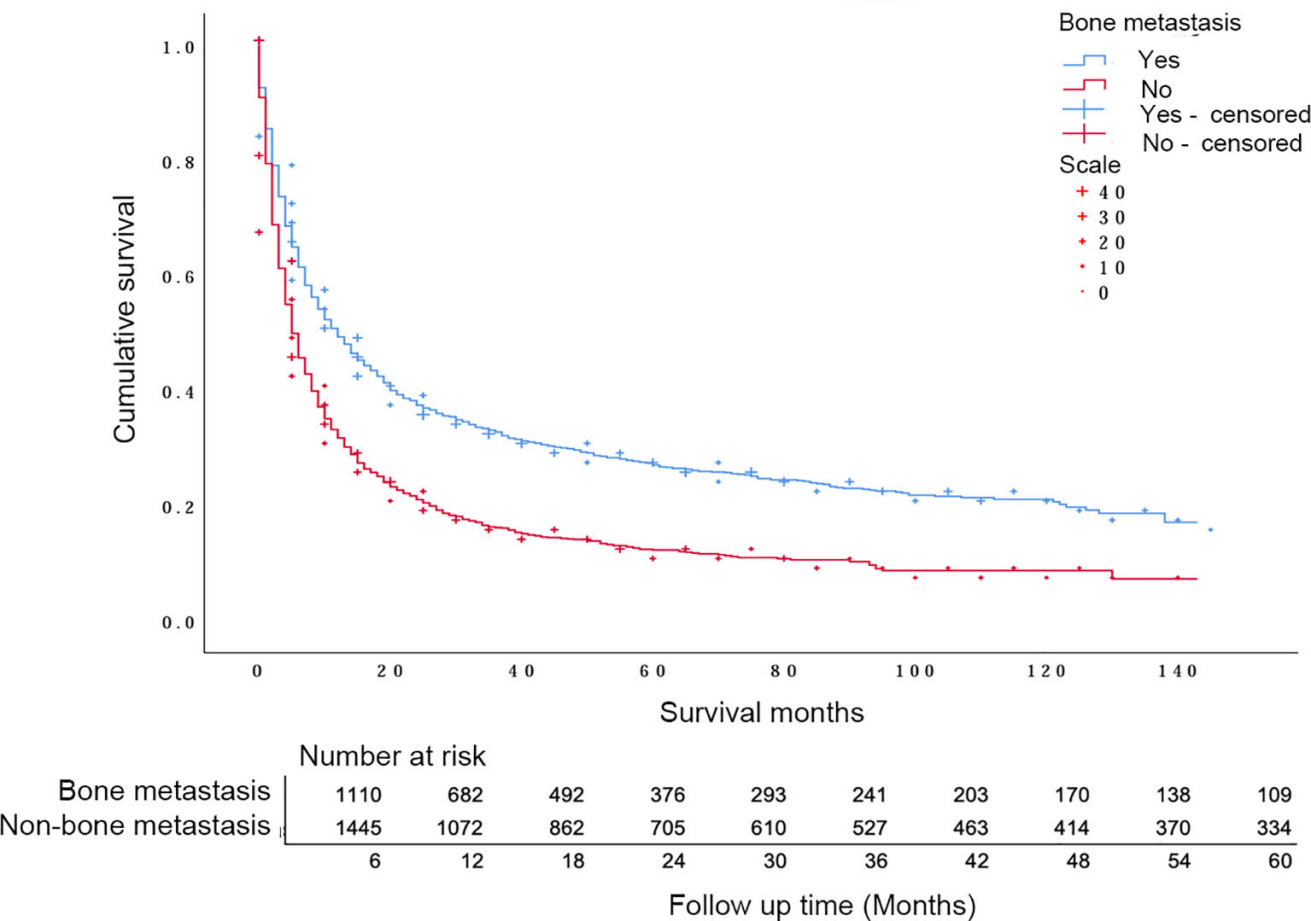
Survival status of patients with melanoma with or without bone metastasis.

The median survival time of the patients was 8 months (95% CI: 7.47-8.53). Specifically, the median survival time for the bone metastasis group was 5 months (95% CI: 4.55-5.45), and for the non-bone metastasis group, it was 12 months (95% CI: 10.61-13.39). The overall 1-year survival rate for patients with bone metastasis was 28.92%, the 3-year survival rate was 10.01%, and the 5-year survival rate was 4.41%. For patients without bone metastasis, the overall 1-year survival rate was 45.46%, the 3-year survival rate was 23.35%, and the 5-year survival rate was 14.16%. There was a statistically significant difference in the overall survival rates between the two groups (χ2 = 188.74, P < 0.001) (**Figure 1**).

### 3.3 Prognostic factors affecting melanoma bone metastasis and construction of the nomogram

Based on the aforementioned results, patients with melanoma accompanied by bone metastasis have a significantly poorer prognosis compared to those without bone metastasis. We further analyzed the prognostic factors affecting patients with melanoma and constructed a prognostic prediction model for bone metastasis of melanoma. A total of 2358 patients with bone metastasis obtained from the SEER database were randomly assigned in a 7:3 ratio to the training cohort (n=1652) and the internal validation cohort (n=706). Additionally, 152 patients with bone metastasis from Fujian Cancer Hospital served as the external validation cohort.

#### 3.3.1 Univariate and multivariate cox regression analysis of prognostic factors for melanoma bone metastasis patients

Univariate Cox analysis may not fully consider all potential influencing factors, while multivariate Cox analysis can more accurately reveal the relationships between variables by considering multiple variables simultaneously and adjusting for the effects of confounding factors. Therefore, to ensure that no potentially significant data is omitted, we included all variables with P < 0.5 from the univariate analysis in the multivariate Cox regression analysis. The multivariate Cox regression analysis indicated that Age, AJCC T stage, AJCC N stage, Surg Prim, Surg or Rad Seq, Systemic and Sur Seq, Ulceration, DX Brain, DX Liver, DX lung, Tumor number, First malignant primary, Marital status, and Urban are independent prognostic factors for patients with melanoma (P<0.05) (**Supplementary Table 3**).

#### 3.3.2 Construction of the nomogram

**Figure 2:**
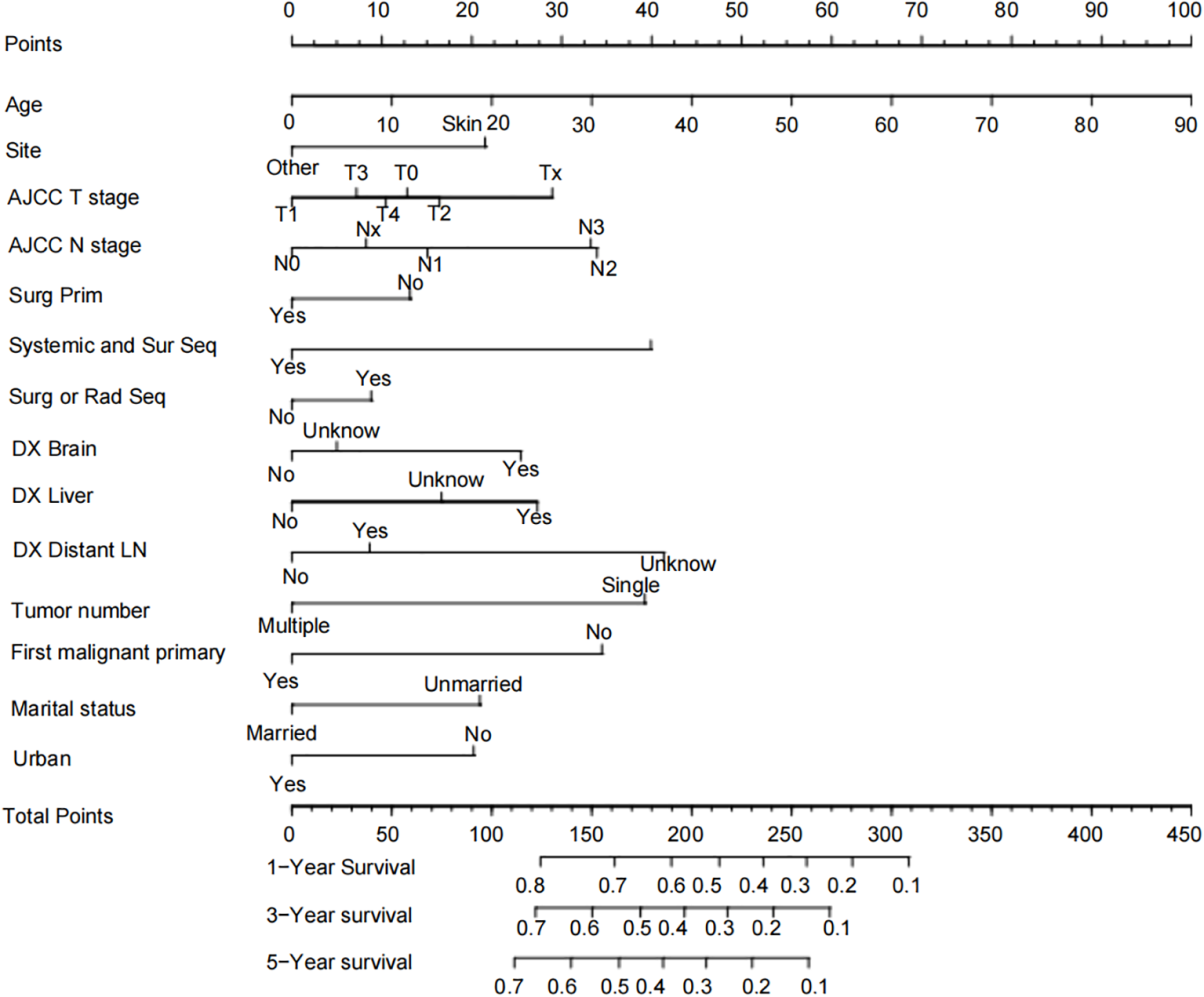
Nomogram prognostic chart for patients with melanoma bone metastasis.

Based on the independent risk factors identified by the multivariate Cox regression, we constructed a Nomogram for the prognosis of bone metastasis in melanoma using the independent risk factors Age, Site, AJCC T stage, AJCC N stage, Surg Prim, Systemic and Sur Seq, Surg or Rad Seq, DX Brain, DX Liver, DX Distant LN, Tumor number, First malignant primary, Marital status, and Urban (**Figure 2**). In the model, each influential factor is assigned a score based on the contribution of each factor to the outcome variable (the magnitude of the regression coefficient) for each value level. The scores are then summed to obtain a total score, with a higher total score being associated with a poorer prognosis. The exact point assignment for each factor and the survival probabilities corresponding to the total points are shown in **Figure 2**. The mortality rate of patients with bone metastasis from melanoma varies from 10% to 80%, and most patients have a total score ranging from 100 to 310. From the Nomogram, it is observed that Age has the greatest predictive contribution to prognosis, with older age correlating with poorer prognosis; AJCC T stage, AJCC N stage, Systemic and Sur Seq, DX Brain, DX Liver, DX Distant LN, Tumor number, and First malignant primary have a moderate predictive contribution to prognosis; Site, Surg Prim, Surg or Rad Seq, Marital status, and Urban have a smaller predictive contribution to prognosis.

#### 3.3.3 Predictive ability of the nomogram prognostic model

**Figure 3:**
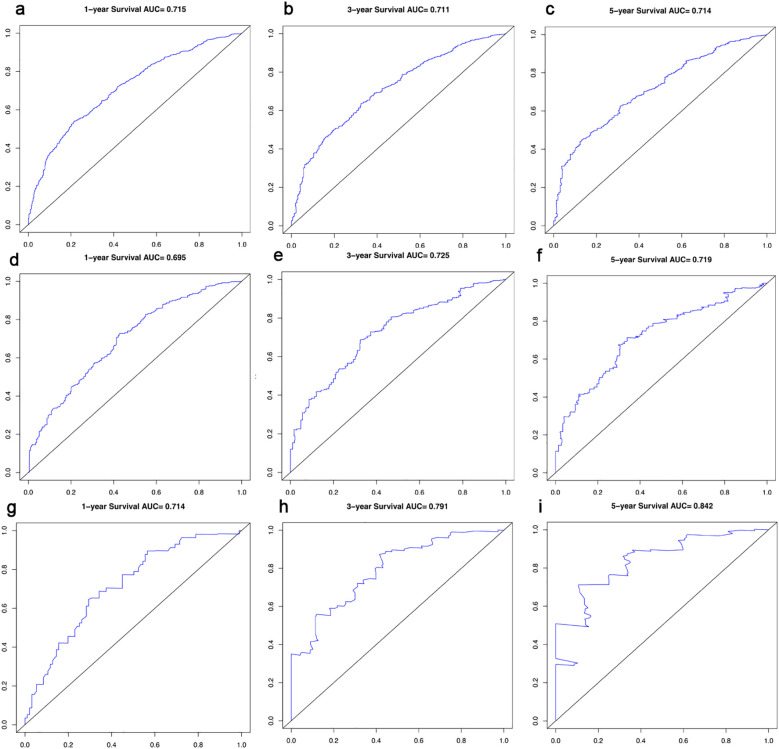
ROC Curves and AUCs for 1-, 3- and 5-year survival in the training cohort **(a-c)**, in the internal validation cohort **(d-f)** and in the external validation cohort **(g-i)**.

**Figure 4:**
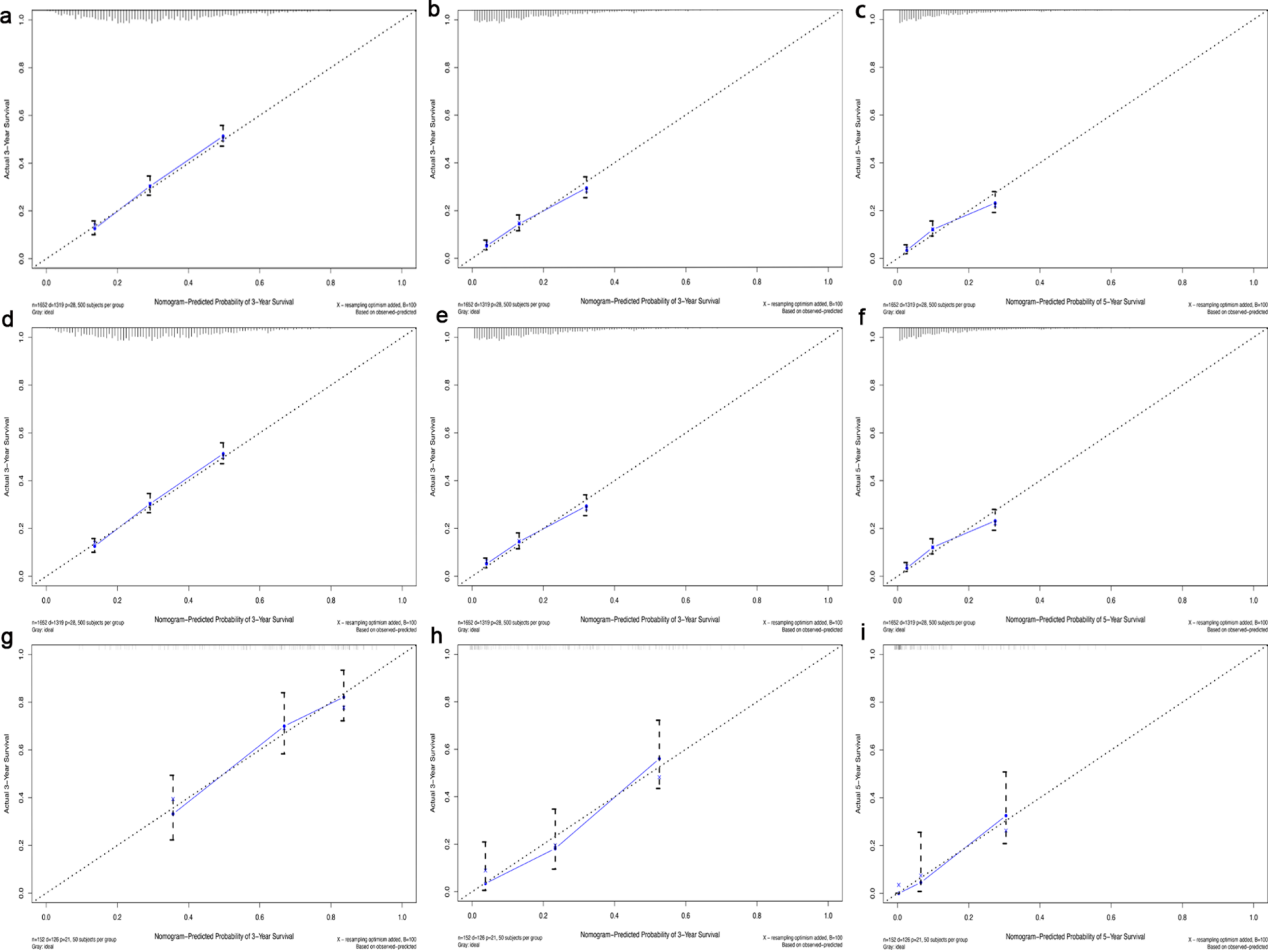
Calibration curve for 1-, 3- and 5-year survival in the training cohort **(a-c)**, in the internal validation cohort **(d-f)** and in the external validation cohort **(g-i)**. The dotted line represented predicted overall survival, and the blue line represented actual overall survival in the cohort.

In this study, the predictive accuracy of the Nomogram chart was assessed using the c-index and AUC (**Figure 3**), and the calibration of the Nomogram chart was evaluated using calibration curves (**Figure 4**). The AUCs for the 1-year, 3-year, and 5-year OS rates in the training cohort were 0.715, 0.711, and 0.714, respectively, with a c-index of 0.656; further statistics for the internal validation cohort showed AUCs of 0.695, 0.725, and 0.719 for the 1-year, 3-year, and 5-year OS rates, respectively, with a c-index of 0.650; the external validation cohort demonstrated AUCs of 0.714, 0.791, and 0.842 for the 1-year, 3-year, and 5-year OS rates, respectively, with a c-index of 0.710. This indicates that the Nomogram chart has excellent predictive power. The calibration curves for the training and validation cohorts respectively displayed the consistency between the observed and predicted 1-year, 3-year, and 5-year OS rates.

## 4 Discussion

The skeleton is the third most common site for metastasis of many solid tumors, including melanoma (4). A study by Zekri et al. showed that the incidence of bone metastasis in their study population reached 17.2% between 2000 and 2008 (6). Another survey conducted by Wilson et al., covering data from 2002 to 2017, recorded an even higher bone metastasis detection rate of up to 43% (8). However, when this study analyzed cases of melanoma registered in the U.S. SEER database from 2000 to 2021, the detection rate of bone metastasis was found to be only 0.9%, significantly lower than the figures reported in the aforementioned literature. This significant discrepancy may be attributed to the fact that the SEER database primarily focuses on the U.S. population, with a predominance of cutaneous melanoma, and that patients are often in the early stages, thereby affecting the statistics of the incidence of bone metastasis.

Existing studies have shown that once melanoma metastasizes to the skeleton, it indicates a poor prognosis, and when the skeleton is the first site of metastasis, the prognosis for patients is worse than for those with initial metastasis to other sites (8). Previous data indicated that before 2011, the median overall survival (mOS) for melanoma patients with bone metastasis was about 4 months (4, 9), in recent years, with the emergence of new therapeutic drugs such as ipilimumab, nivolumab, dabrafenib, and trametinib, there has been an improvement in patient survival, for instance, the mOS reported by Wilson et al. in a certain year has been extended to 9 months (8). Although the SEER database does not directly report the use of immune checkpoint inhibitors, considering that the majority of patients in the United States have cutaneous melanoma, immune checkpoint inhibitors should play a significant role in the treatment of melanoma. This study analyzed patients with bone metastasis of melanoma registered in the SEER database from 2000 to 2021 and found that the mOS was 5 months, a figure that lies between the reports of the two aforementioned time points, reflecting the positive impact of therapeutic advancements on patient survival. We further explored the prognostic factors affecting the overall survival (OS) of patients with bone metastasis and found that older age, higher N stage, and the presence of liver, brain, or distant lymph node metastasis all lead to a poorer prognosis, findings that are consistent with previous literature (7, 10, 11).

Surgery is a classic treatment for melanoma and is widely recognized for its curative potential (12). For patients with higher postoperative staging, adjuvant therapy after surgery can reduce the tumor recurrence rate and extend overall survival (13, 14). This is consistent with our retrospective findings, which found that melanoma patients who undergo systemic therapy during the perioperative period after the surgical removal of the primary lesion, even if bone metastasis occurs later, can still benefit from survival. Although it is generally believed that postoperative adjuvant radiotherapy for patients with positive surgical margins or lymph nodes helps to reduce the risk of recurrence and extend OS (15, 16). However, our retrospective study revealed a different perspective: in the setting of neoadjuvant or adjuvant radiotherapy, such treatment may emerge as a factor associated with poorer prognosis once melanoma patients develop bone metastases. This finding echoes the report by Zhou et al., who pointed out that adjuvant radiotherapy is a risk factor in the prognosis of patients with primary melanoma of the vulva (17). The underlying mechanism of this phenomenon is complex and not yet fully elucidated, and further in-depth exploration is urgently needed.

We are aware that with the rapid development of melanoma drugs, patients with cutaneous melanoma, due to their high tumor mutational burden and a high proportion of BRAF mutations (at least 50%) (18, 19), have shown a more favorable prognosis compared to other subtypes. However, a thought-provoking finding in this study is that when cutaneous melanoma metastasizes to the bones, this advantage turns into a factor for poor prognosis. We speculate that this may be related to the fact that patients with this subtype often develop bone metastasis after receiving immunotherapy or targeted therapy, and the drug-resistant lesions post-treatment may lead to a deterioration in overall prognosis. To verify this hypothesis, more prospective studies are particularly necessary. Additionally, this study also revealed another interesting phenomenon: a single tumor lesion is actually a factor for poor prognosis. This may seem paradoxical but could reflect the clinical practice of neglecting single tumor lesions, while multiple tumor lesions, due to more pronounced symptoms, prompt patients and physicians to be more vigilant. However, we also recognize that there should be a limit to the “number of tumors” here, as an excessive tumor burden can also adversely affect prognosis.

When exploring the impact of patient characteristics on prognosis, we observed a heartwarming and positive finding: marriage has become a protective factor for melanoma patients with bone metastasis. This is consistent with previous studies (20), suggesting that the support of a spouse may improve the patient’s prognosis by detecting suspicious lesions earlier, promoting regular or early screening, and providing substantial help during the treatment process. At the same time, living in urban areas has also been confirmed as one of the protective factors, which may be related to the fact that urban patients have more convenient access to high-quality medical resources, receive cutting-edge treatments, and have higher levels of education and better treatment compliance (20).

After a thorough review and analysis of our institutional data on melanoma patients with bone metastases, we successfully performed external validation that confirmed the robust predictive performance of the nomogram developed in this study. The 1-year OS AUC in the external-validation cohort was comparable to those in both the training and internal-validation cohorts, whereas the 3- and 5-year OS AUCs and the c-index were even higher, suggesting that the model has fortuitously captured more generalizable patterns and is better suited to predicting outcomes in real-world melanoma patients with bone metastases. These findings provide an important reference for clinicians when deciding the optimal timing for bone ECT surveillance. Currently, there is a lack of systemic treatment measures for bone metastasis, but bisphosphonates or RANKL inhibitors can be given to reduce the destruction of osteoclasts, promote the secretion of osteoblast factors, and reduce the risk of osteolytic damage (21–23). At the same time, for local compression symptoms caused by bone metastasis, a comprehensive treatment strategy combining radiotherapy with analgesics can significantly improve the patient’s quality of life and may improve their prognosis (6).

However, this study also has some limitations, including potential selection bias as a retrospective study, the absence of key prognostic data, and the limitation of staging analysis due to the high proportion of Tx and Nx patients in the SEER database. Meanwhile, before modeling in this study, the correlation diagnosis of all covariates was not conducted. Potential collinearity may limit the interpretation of individual coefficients. Therefore, we look forward to further validating and optimizing the predictive ability of this prognostic nomogram through larger-scale, more prospective studies in the future, to better serve the clinical diagnosis and treatment of melanoma bone metastasis patients.

## Data Availability

The raw data supporting the conclusions of this article will be made available by the authors, without undue reservation.
