# Supplementary material for: Survival nomogram for melanoma with bone metastasis based on the SEER database and an external validation cohort
Source: Front Oncol. 2025 Nov 6;15:1680191. doi: 10.3389/fonc.2025.1680191 (PMC12630995; doi:10.3389/fonc.2025.1680191)
Supplement: Supplementary file 1 [file Table1.docx]

Supplementary Table 1. Basic Characteristics of Patients with melanoma with or without Bone Metastasis after Propensity Matching.

|  | Without bone metastasis (n=2358) | Bone metastasis (n=2358) | P |
| --- | --- | --- | --- |
| Survival status |  |  | <0.001 |
| Alive | 754 (32.0) | 465 (19.7) |  |
| Dead | 1604 (68.0) | 1893 (80.3) |  |
| Age | 65.90 (14.51) | 65.23 (14.25) | 0.109 |
| Sex |  |  | 0.874 |
| Male | 1635 (69.3) | 1641 (69.6) |  |
| Fmale | 723 (30.7) | 717 (30.4) |  |
| Race |  |  | 0.247 |
| White | 2267 (96.1) | 2256 (95.7) |  |
| Black | 30 (1.3) | 47 (2.0) |  |
| American Indian or Alaska Native | 7 (0.3) | 7 (0.3) |  |
| Asian or Pacific Islander | 54 (2.3) | 48 (2.0) |  |
| Site |  |  | 0.290 |
| Skin | 2174 (92.2) | 2194 (93.0) |  |
| Other | 184 (7.8) | 164 (7.0) |  |
| AJCC T stage |  |  | 0.246 |
| T0 | 364 (15.4) | 352 (14.9) |  |
| T1 | 46 (2.0) | 49 (2.1) |  |
| T2 | 37 (1.6) | 40 (1.7) |  |
| T3 | 65 (2.8) | 58 (2.5) |  |
| T4 | 108 (4.6) | 146 (6.2) |  |
| Tx | 1738 (73.7) | 1713 (72.6) |  |
| AJCC N stage |  |  | 0.235 |
| N0 | 387 (16.4) | 351 (14.9) |  |
| N1 | 179 (7.6) | 215 (9.1) |  |
| N2 | 40 (1.7) | 34 (1.4) |  |
| N3 | 64 (2.7) | 63 (2.7) |  |
| N4 | 1688 (71.6) | 1695 (71.9) |  |
| Surg Prim |  |  | <0.001 |
| No | 1903 (80.7) | 1710 (72.5) |  |
| Yes | 455 (19.3) | 648 (27.5) |  |
| Scope Reg LN Sur |  |  | <0.001 |
| 1-3 | 26 (1.1) | 59 (2.5) |  |
| ≥4 | 144 (6.1) | 55 (2.3) |  |
| None | 50 (2.1) | 32 (1.4) |  |
| Unknow | 2138 (90.7) | 2212 (93.8) |  |
| Surg Oth Reg or Dis |  |  | 0.111 |
| No | 2328 (98.7) | 2340 99.2) |  |
| Yes | 30 (1.3) | 18 (0.8) |  |
| Surg or Rad Seq |  |  | 0.039 |
| No | 1874 (79.5) | 1931 (81.9) |  |
| Yes | 484 (20.5) | 427 (18.1) |  |
| Chemotherapy |  |  | 0.299 |
| No | 1746 (74.0) | 1778 (75.4) |  |
| Yes | 612 (26.0) | 580 (24.6) |  |
| Radiation |  |  | 0.001 |
| No | 51 (2.2) | 21 (0.9) |  |
| Yes | 989 (41.9) | 1022 (43.3) |  |
| Unknow | 1318 (55.9) | 1315 (55.8) |  |
| Systemic and Sur Seq |  |  | <0.001 |
| No | 1529 (64.8) | 1692 (71.8) |  |
| Yes | 829 (35.2) | 666 (28.2) |  |
| Ulceration |  |  | <0.001 |
| No | 292 (12.4) | 408 (17.3) |  |
| Yes | 286 (12.1) | 371 (15.7) |  |
| Unknow | 1780 (75.5) | 1579 (67.0) |  |
| DX Brain |  |  | <0.001 |
| No | 1531 (64.9) | 1536 (65.1) |  |
| Yes | 811 (34.4) | 752 (31.9) |  |
| Unknow | 16 (0.7) | 70 (3.0) |  |
| DX Liver |  |  | <0.001 |
| No | 1467 (62.2) | 1193 (50.6) |  |
| Yes | 826 (35.0) | 1104 (46.8) |  |
| Unknow | 65 (2.8) | 61 (2.6) |  |
| DX Lung |  |  | 0.247 |
| No | 1005 (42.6) | 953 (40.4) |  |
| Yes | 1280 (54.3) | 1337 (56.7) |  |
| Unknow | 73 (3.1) | 68 (2.9) |  |
| DX Distant LN |  |  | <0.001 |
| No | 861 (36.5) | 795 (33.7) |  |
| Yes | 287 (12.2) | 477 (20.2) |  |
| Unknow | 1210 (51.3) | 1086 (46.1) |  |
| DX Other |  |  | <0.001 |
| No | 806 (34.2) | 730 (31.0) |  |
| Yes | 387 (16.4) | 592 (25.1) |  |
| Unknow | 1165 (49.4) | 1036 (43.9) |  |
| Tumor number |  |  | 0.619 |
| Single | 1597 (67.7) | 1580 (67.0) |  |
| Multiple | 761 (32.3) | 778 (33.0) |  |
| First malignant primary |  |  | 1 |
| No | 627 (26.6) | 628 (26.6) |  |
| Yes | 1731 (73.4) | 1730 (73.4) |  |
| Marital status |  |  | 0.185 |
| Married | 1032 (43.8) | 986 (41.8) |  |
| Unmarried | 1326 (56.2) | 1372 (58.2) |  |
| Urban |  |  | 0.896 |
| No | 302 (12.8) | 298 (12.6) |  |
| Yes | 2056 (87.2) | 2060 (87.4) |  |

Supplementary Table 2. Comparison of baseline characteristics between Seer Cohort and External Validation Cohort.

|  | External Validation Cohort  (n=152) | Seer Cohort  (n=2358) | p |
| --- | --- | --- | --- |
| Age | 65.23 (14.25) | 56.15 (13.25) | <0.001 |
| Site |  |  |  |
| Skin | 30 (19.7) | 2194 (93.0) | <0.001 |
| Other | 122 (80.3) | 164 (7.0) |  |
| AJCC T stage |  |  | <0.001 |
| T0 | 2 (1.3) | 352 (14.9) |  |
| T1 | 3 (2.0) | 49 (2.1) |  |
| T2 | 7 (4.6) | 40 (1.7) |  |
| T3 | 12 (7.9) | 58 (2.5) |  |
| T4 | 32 (21.1) | 146 (6.2) |  |
| Tx | 96 (63.2) | 1713 (72.6) |  |
| AJCC N stage |  |  | <0.001 |
| N0 | 41 (27.0) | 351 (14.9) |  |
| N1 | 9 (5.9) | 215 (9.1) |  |
| N2 | 8 (5.3) | 34 (1.4) |  |
| N3 | 33 (21.7) | 63 (2.7) |  |
| N4 | 61 (40.1) | 1695 (71.9) |  |
| Surg Prim |  |  | <0.001 |
| No | 66 (43.4) | 1710 (72.5) |  |
| Yes | 86 (56.6) | 648 (27.5) |  |
| Scope Reg LN Sur |  |  | <0.001 |
| <4 | 118 (77.6) | 59 (2.5) |  |
| ≥4 | 34 (22.4) | 55 (2.3) |  |
| None | 0 (0) | 32 (1.4) |  |
| Unknow | 0 (0) | 2212 (93.8) |  |
| Surg or Rad Seq |  |  | <0.001 |
| No | 142 (93.4) | 1931 (81.9) |  |
| Yes | 10 (6.6) | 427 (18.1) |  |
| Chemotherapy |  |  | <0.001 |
| No | 71 (46.7) | 1778 (75.4) |  |
| Yes | 81 (53.3) | 580 (24.6) |  |
| Radiation |  |  | <0.001 |
| No | 97 (63.8) | 21 (0.9) |  |
| Yes | 55 (36.2) | 1022 (43.3) |  |
| Unknow | 0 (0) | 1315 (55.8) |  |
| Systemic and Sur Seq |  |  |  |
| No | 115 (75.7) | 1692 (71.8) | 0.299 |
| Yes | 37 (24.3) | 666 (28.2) |  |
| Ulceration |  |  | 0.048 |
| No | 15 (9.9) | 408 (17.3) |  |
| Yes | 23 (15.1) | 371 (15.7) |  |
| Unknow | 114 (75.0) | 1579 (67.0) |  |
| DX Brain |  |  | 0.087 |
| No | 105 (69.1) | 1536 (65.1) |  |
| Yes | 47 (30.9) | 752 (31.9) |  |
| Unknow | 0 (0.0) | 70 (3.0) |  |
| DX liver |  |  | 0.022 |
| No | 67 (44.1) | 1193 (50.6) |  |
| Yes | 85 (55.9) | 1104 (46.8) |  |
| Unknow | 0 (0) | 61 (2.6) |  |
| DX lung |  |  | 0.264 |
| No | 62 (40.8) | 953 (40.4) |  |
| Yes | 89 (58.6) | 1337 (56.7) |  |
| Unknow | 1 (0.7) | 68 (2.9) |  |
| DX Distant LN |  |  | <0.001 |
| No | 59 (38.8) | 795 (33.7) |  |
| Yes | 93 (61.2) | 477 (20.2) |  |
| Unknow | 0 (0) | 1086 (46.1) |  |
| DX Other |  |  | <0.001 |
| No | 68 (44.7) | 730 (31.0) |  |
| Yes | 84 (55.3) | 592 (25.1) |  |
| Unknow | 0 (0) | 1036 (43.9) |  |
| Multi primary |  |  | <0.001 |
| No | 143 (94.1) | 1580 (67.0) |  |
| Yes | 9 (5.9) | 778 (33.0) |  |
| First malignant primary |  |  | <0.001 |
| No | 7 (4.6) | 628 (26.6) |  |
| Yes | 145 (95.4) | 1730 (73.4) |  |
| Marital status |  |  | <0.001 |
| Married | 17 (11.2) | 986 (41.8) |  |
| Unmarried | 135 (88.8) | 1372 (58.2) |  |
| Urban |  |  | <0.001 |
| No | 102 (67.1) | 298 (12.6) |  |
| Yes | 50 (32.9) | 2060 (87.4) |  |

Supplementary Table 3. Univariate and Multivariate Cox Regression Analysis of Prognostic Factors for Bone Metastasis in melanoma.

|  | Univariate Cox Regression | | | Multivariate Cox Regression | |
| --- | --- | --- | --- | --- | --- |
|  | HR (95%CI) | | Z (P) | HR (95%CI) | Z (P) |
| Age | 1.009 (1.005-1.013) | 4.436 (<0.001) | | 1.012 (1.008-1.017) | 5.760 (<0.001) |
| Sex |  |  | |  |  |
| Male | Reference |  | |  |  |
| Fmale | 0.969 (0.862-1.089) | -0.532 (0.595) | |  |  |
| Race |  |  | |  |  |
| White | Reference | - | | Reference | - |
| Black | 1.010 (0.694-1.469) | 0.050 (0.960) | | 1.149 (0.783-1.686) | 0.712 (0.477) |
| American Indian or Alaska Native | 0.415 (0.104-1.661) | -1.243 (0.214) | | 0.428 (0.106-1.729) | -1.192 (0.233) |
| Asian or Pacific Islander | 1.007 (0.688-1.475) | 0.038 (0.970) | | 0.921 (0.624-1.359) | -0.415 (0.678) |
| Site |  |  | |  |  |
| Skin | Reference | - | | Reference | - |
| Other | 0.849 (0.686-1.049) | -1.52 (0.129) | | 0.749 (0.590-0.951) | -2.369 (0.018) |
| AJCC T stage |  |  | |  |  |
| T0 | Reference | - | | Reference | - |
| T1 | 0.894 (0.612-1.307) | -0.576 (0.564) | | 0.932 (0.613-1.418) | -0.327 (0.743) |
| T2 | 0.889 (0.580-1.364) | -0.538 (0.590) | | 1.078 (0.674-1.725) | 0.315 (0.753) |
| T3 | 0.867 (0.620-1.212) | -0.836 (0.403) | | 0.964 (0.666-1.396) | -0.194 (0.846) |
| T4 | 0.861 (0.677-1.095) | -1.219 (0.223) | | 0.989 (0.731-1.339) | -0.071 (0.944) |
| Tx | 0.829 (0.718-0.957) | -2.561 (0.010) | | 1.286 (1.066-1.551) | 2.623 (0.009) |
| AJCC_N_stage |  |  | |  |  |
| N0 | Reference | - | | Reference | - |
| N1 | 1.083 (0.878-1.335) | 0.746 (0.456) | | 1.181 (0.947-1.473) | 1.479 (0.139) |
| N2 | 1.165 (0.765-1.774) | 0.710 (0.478) | | 1.538 (0.989-2.392) | 1.911 (0.056) |
| N3 | 1.227 (0.882-1.705) | 1.216 (0.224) | | 1.525 (1.069-2.175) | 2.327 (0.020) |
| Nx | 0.923 (0.798-1.068) | -1.078 (0.281) | | 1.125 (0.920-1.377) | 1.146 (0.252) |
| Surg Prim |  |  | |  |  |
| No | Reference | - | | Reference | - |
| Yes | 0.688 (0.608-0.779) | -5.913 (<0.001) | | 0.800 (0.666-0.961) | -2.358 (0.017) |
| Scope Reg LN Sur |  |  | |  |  |
| 1-3 | Reference | - | | Reference | - |
| ≥4 | 0.995 (0.597-1.657) | -0.021 (0.984) | | 0.831 (0.491-1.407) | -0.689 (0.491) |
| None | 1.408 (0.974-2.034) | 1.819 (0.069) | | 0.918 (0.624-1.352) | -0.432 (0.666) |
| Unknow | 1.040 (0.597-1.810) | 0.138 (0.890) | | 0.807 (0.458-1.422) | -0.743 (0.458) |
| Surg Oth Reg or Dis |  |  | |  |  |
| No | Reference | - | | Reference | - |
| Yes | 0.488 (0.243-0.977) | -2.026 (0.043) | | 0.628 (0.310-1.271) | -1.295 (0.195) |
| Surg or Rad Seq |  |  | |  |  |
| No | Reference | - | | Reference | - |
| Yes | 0.846 (0.734-0.976) | -2.298 (0.022) | | 1.242 (1.010-1.528) | 2.057 (0.040) |
| Chemotherapy |  |  | |  |  |
| No | Reference | - | | Reference | - |
| Yes | 0.911 (0.806-1.03) | -1.492 (0.136) | | 0.910 (0.795-1.042) | -1.370 (0.171) |
| Radiation |  |  | |  |  |
| No | Reference | - | | Reference | - |
| Yes | 0.629 (0.370-1.070) | -1.711 (0.087) | | 0.594 (0.340-1.038) | -1.829 (0.067) |
| Unknow | 0.616 (0.363-1.045) | -1.797 (0.072) | | 0.697 (0.402-1.209) | -1.286 (0.199) |
| Systemic and Sur Seq |  |  | |  |  |
| No | Reference | - | | Reference | - |
| Yes | 0.552 (0.486-0.626) | -9.25 (<0.001) | | 0.617 (0.522-0.731) | -5.616 (<0.001) |
| Ulceration |  |  | |  |  |
| No | Reference | - | | Reference | - |
| Yes | 1.125 (0.928-1.363) | 1.200 (0.230) | | 1.213 (0.978-1.505) | 1.751 (0.080) |
| Unknow | 1.304 (1.123-1.514) | 3.478 (0.002) | | 1.111 (0.937-1.318) | 1.216 (0.224) |
| DX Brain |  |  | |  |  |
| No | Reference | - | | Reference | - |
| Yes | 1.355 (1.207-1.522) | 5.152 (<0.001) | | 1.332 (1.168-1.519) | 4.270 (<0.001) |
| Unknow | 1.262 (0.915-1.740) | 1.419 (0.156) | | 0.986 (0.665-1.460) | -0.073 (0.942) |
| DX Liver |  |  | |  |  |
| No | Reference | - | | Reference | - |
| Yes | 1.420 (1.273-1.586) | 6.259 (<0.001) | | 1.373 (1.222-1.544) | 5.311 (<0.001) |
| Unknow | 1.512 (1.075-2.125) | 2.379 (0.017) | | 1.211 (0.805-1.823) | 0.919 (0.358) |
| DX lung |  |  | |  |  |
| No | Reference | - | | Reference | - |
| Yes | 1.377 (1.231-1.541) | 5.579 (<0.001) | | 1.121 (0.990-1.269) | 1.804 (0.072) |
| Unknow | 1.209 (0.883-1.656) | 1.183 (0.237) | | 0.859 (0.599-1.231) | -0.829 (0.407) |
| DX Distant LN |  |  | |  |  |
| No | Reference | - | | Reference | - |
| Yes | 1.230 (1.045-1.447) | 2.492 (0.013) | | 1.127 (0.946-1.343) | 1.340 (0.180) |
| Unknow | 1.498 (1.322-1.697) | 6.342 (<0.001) | | 1.435 (1.048-1.965) | 2.252 (0.024) |
| DX Other |  |  | |  |  |
| No | Reference | - | | Reference | - |
| Yes | 1.220 (1.046-1.425) | 2.524 (0.012) | | 0.952 (0.803-1.129) | -0.566 (0.572) |
| Unknow | 1.472 (1.292-1.677) | 5.804 (<0.001) | | 1.262 (0.901-1.769) | 1.354 (0.176) |
| Tumor number |  |  | |  |  |
| Single | Reference | - | | Reference | - |
| Multiple | 0.894 (0.797-1.003) | -1.906 (0.057) | | 0.608 (0.474-0.780) | -3.915 (<0.001) |
| First malignant primary |  |  | |  |  |
| No | Reference | - | | Reference | - |
| Yes | 0.942 (0.835-1.062) | -0.975 (0.330) | | 0.632 (0.487-0.819) | -3.464 (<0.001) |
| Marital status |  |  | |  |  |
| Unmarried | Reference | - | | Reference | - |
| Married | 0.819 (0.734-0.913) | -3.603 (<0.001) | | 0.772 (0.690-0.865) | -4.472 (<0.001) |
| Urban |  |  | |  |  |
| No | Reference | - | | Reference | - |
| Yes | 0.850 (0.723-1.000) | -1.958 (0.050) | | 0.770 (0.653-0.907) | -3.116 (0.002) |
